# Down-Regulation of Phosphoribosyl Pyrophosphate Synthetase 1 Inhibits Neuroblastoma Cell Proliferation

**DOI:** 10.3390/cells8090955

**Published:** 2019-08-22

**Authors:** Jifu Li, Junhong Ye, Shunqin Zhu, Hongjuan Cui

**Affiliations:** 1 State Key Laboratory of Silkworm Genome Biology, College of Biotechnology, Southwest University, Chongqing 400715, China; 2 Biological Science Research Center, Southwest University, Chongqing 400715, China; 3 School of Life Sciences, Southwest University, Chongqing 400715, China; 4 Engineering Research Center for Cancer Biomedical and Translational Medicine, Southwest University, Chongqing 400715, China; 5 Chongqing Engineering and Technology Research Center for Silk Biomaterials and Regenerative Medicine, Southwest University, Chongqing 400715, China; 6 Cancer Center, Medical Research Institute, Southwest University, Chongqing 400715, China

**Keywords:** phosphoribosyl pyrophosphate synthetase 1 (PRPS1), neuroblastoma, cell proliferation

## Abstract

Phosphoribosyl pyrophosphate synthetase 1 (PRPS1) is a key enzyme in de novo nucleotide synthesis and nucleotide salvage synthesis pathways that are critical for purine and pyrimidine biosynthesis. Abnormally high expression of PRPS1 can cause many diseases, including hearing loss, hypotonia, and ataxia, in addition to being associated with neuroblastoma. However, the role of PRPS1 in neuroblastoma is still unclear. In this study, we found that PRPS1 was commonly expressed in neuroblastoma cells and was closely related to poor prognosis for cancer. Furthermore, down-regulation of PRPS1 inhibited neuroblastoma cell proliferation and tumor growth in vitro and in vivo via disturbing DNA synthesis. This study provides new insights into the treatment of neuroblastoma patients and new targets for drug development.

## 1. Introduction

Neuroblastoma is one of the most common types of solid tumor and exhibits high malignancy rates in children [1,2,3,4]. The tumors often occur in nerve tissues in the adrenal medulla, thoracic cavity, retroperitoneum, and neck, among other areas [5]. Clinically, neuroblastoma has a poor prognosis, easily metastasizes, recurs frequently, and is very heterogeneous [6,7,8]. Neuroblastoma can be divided into three types based on the degree of differentiation: undifferentiated, poorly differentiated, and differentiated. Besides, a small number of neuroblastoma cases can transform into benign tumors spontaneously with little or no systemic treatment. Neuroblastoma is also self-regressing and differentiates in vitro, rendering it a good tumor model to understand the mechanisms of cancer cell proliferation and differentiation and ultimately identify new strategies for clinical treatment [6,9,10,11].

Nucleotides are central to cellular functioning and also serve as cofactors in cellular metabolism and signaling. The ability to alter metabolites to fulfill the biosynthetic and bioenergy demands of cell growth and proliferation is a defining feature of cancer cells. Therefore, understanding how key cellular processes underlying cancer cell growth like nucleotide synthesis become important. Phosphoribosyl pyrophosphate synthetase (PRPS) is an important substrate for de novo nucleotide synthesis and nucleotide salvage synthesis pathways, and the first reaction of the de novo synthesis pathway requires PRPS1 to catalyze. Therefore, PRPS1 is an important rate-limiting enzyme in nucleotide metabolism [12]. The gene-encoding PRPS1 is located on the Xq22-q24 region of the long arm of the X chromosome [13,14]. PRPS1 is closely related to the biosynthesis of adenine triphosphate nucleoside (ATP), which is a substrate for PRPS, and adenine also requires PRPS for biosynthesis [15]. Aberrant expression of PRPS1 has been implicated in many human diseases, including hearing loss, uric acid overproduction, hypotonia, ataxia, neurodevelopment abnormalities, postlingual hearing impairment, X-linked nonsyndromic sensorineural deafness (DFNX-2), Charcot-Marie-Tooth disease-5 (CMTX5), Arts syndrome, prenatal growth restrictions, retinal dystrophy, diabetes insipidus, and white matter disease [16,17,18,19,20,21,22].

PRPS1 has also recently attracted attention for its role in the development of various cancers. PRPS1 may inhibit tumor cell proliferation and differentiation in addition to affecting cell migration. Besides, PRPS1 has important effects on cellular proliferation, clonality, and apoptosis in glioblastoma multiforme (GBM) [23]. Moreover, PRPS1 plays an important role in the recurrence of relapsed acute lymphoblastic leukemia (ALL) [24,25]. Furthermore, miR124 can target PRPS1 and further affect the activity of the pentose phosphate pathway and cellular proliferation in colon cancers [26]. PRPS1 phosphorylation is also associated with poor prognosis in hepatocellular carcinoma, where it has been shown that fructokinase phosphorylates and activates PRPS1 to promote the pentose phosphate pathway and formate hepatocellular carcinoma [27]. Finally, PRPS1 is a direct target of miR-154 in CD133^+^ GBM cells in glioblastoma and can affect cellular proliferation and migration [28].

However, no investigation has been conducted to evaluate the role of PRPS1 in neuroblastoma and the molecular mechanisms underlying this relationship. To address this knowledge gap, we investigated the effects of PRPS1 on the growth and proliferation of neuroblastoma cells in the present study. These experiments indicated that down-regulation of PRPS1 expression could significantly reduce cell growth and proliferation in vivo and in vitro. Moreover, these results show that PRPS1 represents a new target gene for clinical research.

## 2. Materials and Methods

### 2.1. Patient Data Analysis

All analyses of prognosis and cell signaling were conducted online. Data that were used in the analyses included gene expression datasets and patient data that were downloaded from the microarray analysis and visualization platform of the R2: Genomics Analysis and Visualization Platform (http://r2.amc.nl). Besides, Kaplan–Meier and survival curve analyses were conducted using the GraphPad Prism software package (version 6.0, https://www.graphpad.com/).

### 2.2. Cell Culture and Treatment

Human neuroblastoma cell lines SK-N-AS, SK-N-DZ, and SHEP1 were cultured in Dulbecco’s modified Eagle’s medium (DMEM, Thermo Fisher Scientific, Waltham, MA, USA) supplemented with 10% fetal bovine serum (FBS, Thermo Fisher Scientific, Waltham, MA, USA). Media were also supplemented with antibiotics (penicillin and streptomycin) at a 1% concentration (P/S, Thermo Fisher Scientific, Waltham, MA, USA). BE(2)-C cells were cultured in a 1:1 mixture of DMEM and Ham’s nutrient mixture F12 (DMEM/F12, Thermo Fisher Scientific, Waltham, MA, USA) that was also supplemented with 10% FBS and 1% P/S antibiotics. All four cell lines were purchased from the American Type Culture Collection (ATCC; Manassas, VA, USA). The lentiviral packaging cell line 293FT was cultured in DMEM containing 10% FBS, 1% P/S, 0.5 mg/mL G418, 4 mM L-glutamine, 0.1 mM MEM non-essential amino acids, and 1 mM MEM sodium pyruvate. The 293FT cell line, growth media, FBS, and supplemental reagents were obtained from Thermo Fisher Scientific (Waltham, MA, USA). All cells were cultured in a fully humidified incubator at 37 °C with a 5% CO_2_ atmosphere. Cells were photographed using a Nikon TS100 inverted microscope (Nikon Corporation, Tokyo, Japan) at 40× magnification. The Image-Pro Plus 6.0 software program (Media Cybernetics, Inc., Rockville, MD, USA) was used for micrograph analyses.

### 2.3. Vector Construction, Transfection, and Infection

To stimulate down-regulation of PRPS1, the lentiviral constructs pLK0.1-puro-PRPS1si (1#/2#) and negative control (pLK0.1-puro-GFPsi) were used in knockdown experiments. The target sequences of these sites were:

PRPS1si-1#F: CCGGTGGACTTTGCCTTGATTCACACTCGAGTGTGAATCAAGGCAAAGTCCATTTTTG; 

PRPS1si-1#R: AATTCAAAAATGGACTTTGCCTTGATTCACACTCGAGTGTGAATCAAGGCAAAGTCCA; 

PRPS1si-2#F: CCGGGAATCCGTTTCTTACCTATTCCTCGAGGAATAGGTAAGAAACGGATTCTTTTTG;

PRPS1si-2#R: AATTCAAAAAGAATCCGTTTCTTACCTATTCCTCGAGGAATAGGTAAGAAACGGATTC.

The lentiviral constructs were transfected into 293FT packaging cells using the Invitrogen^®^ Lipofectamine^®^ 2000 reagent (Thermo Fisher Scientific, Waltham, MA, USA), according to the manufacturer’s protocol. Virus-containing supernatants were harvested and titred, and the lentivirus was then infected into target cells with 4 µg/mL Polybrene (Santa Cruz Biotechnology, Inc., Dallas, TX, USA). The transfected cells were selected with 2 µg/mL puromycin after the final round of infection (Thermo Fisher Scientific, Waltham, MA, USA) for three days. Drug-resistant cells were subsequently collected, expanded, and identified.

### 2.4. Cell Proliferation Analysis

Cell proliferation was measured using the Cell Counting Kit-8 (CCK-8; Beyotime, shanghai, China) assay. For cell proliferation assays, SHEP1 and BE(2)-C cells were seeded and cultured in six-well culture plates. A total of 1000 cells were then transferred into 200 μL medium and seeded into individual wells of 96-well plates. CCK-8 (20 μL) was added into the medium in each well and then incubated at 37 °C for 2 h every two days. The absorbances of the cultures were measured at a wavelength of 450 nm using a microplate reader (Model 550; Bio-Rad Laboratories, Inc., Hercules, CA, USA), according to the manufacturer’s protocol.

### 2.5. Bromodeoxyuridine (BrdU) Staining

Neuroblastoma cells with GFP or PRPS1 knockdowns were plated at a concentration of 2 × 10^3^ cells per well in 24-well culture plates. After cells were adherent, they were treated with 10 μg/mL bromodeoxyuridine (BrdU; Sigma-Aldrich; Merck KGaA, Darmstadt, Germany) for 30 min. The cells were then washed with phosphate-buffered saline (PBS) three times, fixed for 20 min in 4% paraformaldehyde (PFA), and permeabilized with 1 mol/L HCl. Cells were then blocked with 10% goat serum in PBS for 2 h at room temperature, followed by a primary rat antibody against BrdU (1:200, cat. no. ab6326; Abcam, Cambridge, UK) in blocking buffer for 2 h at 4 °C. The cells were then incubated with the secondary antibody, Alexa FluorR^®^ 594 goat anti-rat IgG (1:2000; H + L; Thermo Fisher Scientific, Waltham, MA, USA) for 1 h at room temperature. Also, DAPI (300 nM; Beyotime, shanghai, China) in PBS was used for nuclear staining. The percentage of BrdU-positive cells was calculated from at least 10 microscopic fields (Olympus CKX41; Olympus Corp., Tokyo, Japan).

### 2.6. Cell Cycle Analysis

A total of 1 × 10^6^ cells were harvested, washed, and fixed with ice-cold 70% ethanol, followed by staining with propidium iodide (PI; BD Biosciences, San Jose, CA, USA) and RNase A for 30 min at room temperature. Cells were then analyzed by flow cytometry (FCM; Accuri C6; BD Biosciences, San Jose, CA, USA), and the sorting data were analyzed with the ModfitLT software package (version 4.1, Verity Software House, Topsham, ME).

### 2.7. Western Blot Analysis

Human neuroblastoma cells or xenograft tumor tissues were harvested, washed, and then suspended in RIPA lysis buffer (Beyotime, shanghai, China). Protein concentrations were determined using an Enhanced BCA protein assay kit (Beyotime, shanghai, China), according to the manufacturer’s protocol. A total of 60 µg of each protein sample was separated on 10% sodium dodecyl sulfate-polyacrylamide gels (SDS-PAGE), transferred to polyvinylidene fluoride (PVDF) membranes (EMD Millipore Corp, Billerica, MA, USA), probed with antibodies, and then visualized with BeyoECL Plus (Beyotime, shanghai, China). The primary antibodies used for Western blot analysis included anti-PRPS1 (1:500; cat. no.sc-100822, Santa Cruz Biotechnology, Inc., Dallas, TX, USA) and anti-α-tubulin (1:2000; cat. no. B-5-1-2; Sigma-Aldrich; Merck KGaA, Darmstadt, Germany). The secondary antibodies included Horseradish peroxidase-conjugated goat anti-mouse (1:20,000; KPL, Inc., Gaithersburg, MD, USA) and rabbit anti-goat (1:20,000; KPL, Inc., Gaithersburg, MD, USA) immunoglobulin G (IgG 1:20,000; KPL, Inc., Gaithersburg, MD, USA).

### 2.8. Quantitative RT-PCR

Total RNA was extracted from human neuroblastoma cell lines using the TriZol method (Thermo Fisher Scientific, Waltham, MA, USA), following the manufacturer’s instructions. RNA detection and cDNA template strand synthesis were then conducted using the M-MLV kit (Promega Corp., Madison, WI, USA), as recommended by the manufacturer’s protocols. Target mRNA transcript levels were determined using SYBR^®^ Green PCR Master Mix (Takara, Shiga, Japan), and qRT-PCR reactions were conducted on a OneStep Plus7500 Real-Time PCR system (Bio-Rad Laboratories, Inc., Hercules, CA, USA) in triplicate. All qRT-PCR reagents were obtained from Promega (Madison, WI, USA), including the GoTaq^®^ qPCR Master Mix kit and GAPDH (glyceraldehyde-3-phosphate dehydrogenase) that was used as an internal reference. qRT-PCR was conducted, following the reagent manufacturer recommendations.

Individual expression values were normalized against the GAPDH control, and relative mRNA expression levels were calculated using the ΔΔCq method. The primers that were used were:

GAPDH RT-F:

5′-AACGGATTTGGTCGTATTGGG-3′;

GAPDH RT-R:

5′-CCTGGAAGATGGTGATGGGAT-3′;

PRPS1 RT-F:

5′-GGCTGACACTTGTGGCACAATC-3′;

PRPS1 RT-R:

5′-GATGCGAGAAATAGCAGGACCG-3′.

### 2.9. Soft Agar Clonogenic Assay

A total of 1000 BE(2)-C cells were mixed with 0.3% Noble agar in growth medium and then plated in six-well plates containing a solidified bottom layer (0.6% Noble agar in growth medium). After 14–21 days of cell growth, colonies were stained with 5 mg/mL MTT (Sigma-Aldrich, Merck KGaA, Darmstadt, Germany), photographed microscopically (Olympus CKX41; Olympus Corp., Tokyo, Japan), and recorded.

### 2.10. Tumor Xenograft Assays

BE(2)-C cells were grown to 70–80% confluency and then trypsinized. Cells (1 × 10^6^ in 200 μL serum-free DMEM) were injected into the flanks of six female non-obese diabetic/severe combined immunodeficient (NOD/SCID) mice (four weeks of age, the average weight of 18 g). Tumor growth was measured every three days after one week using digital calipers, and tumor volume (V) was calculated using the formula: 4/3пr^3^, where ‘r’ is the radius of the tumor. Xenograft tumors were then removed and weighed after 19 weeks of growth. All animal experiments were pre-approved and supervised by the Institutional Animal Care and Use Committees of the Southwest University.

### 2.11. Histology and Immunohistochemistry

The tumors were washed with ice-cold PBS and embedded in paraffin blocks. Tumors were then sectioned at a thickness of 4 µm and stained with hematoxylin and eosin (H&E). The sections were examined using a Nikon 80i light microscope (Nikon, Tokyo, Japan).

### 2.12. Statistical Analyses

All of the experiments were repeated with at least three independent replicates, and data are expressed as the mean ± S.D. (standard deviation). GraphPad Prism 6 (GraphPad Software, Inc., La Jolla, CA, USA) was used for statistical analyses. Statistical significance was determined using a two-tailed unpaired Student’s *t*-test. Statistically significant differences were considered at the *p* < 0.05 level.

## 3. Results

### 3.1. PRPS1 is Associated with Poor Neuroblastoma Patient Progress and is Commonly Expressed in Neuroblastoma Cells

**Figure 1:**
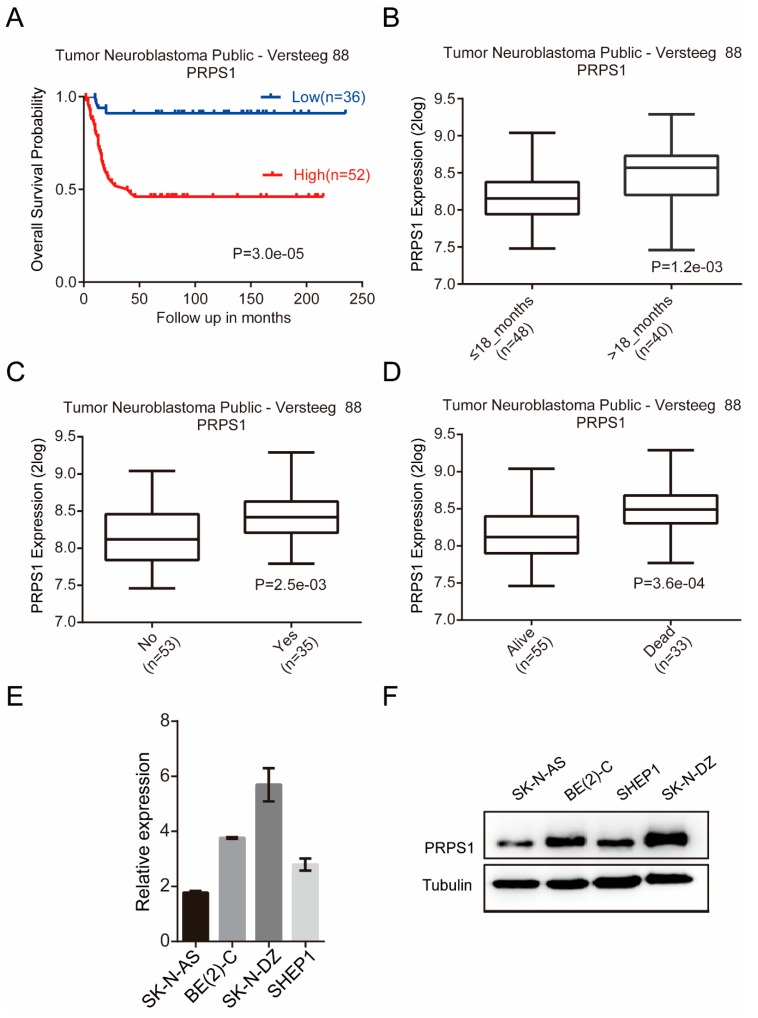
High expression of phosphoribosyl pyrophosphate synthetase 1 (PRPS1) predicts poor prognosis of patients with neuroblastoma and is commonly expressed in neuroblastoma cells. (**A**) The relationship between PRPS1 expression and the overall survival probability, based on data from the Tumor Neuroblastoma Public-Versteeg 88 database within the R2 platform. (**B**) The expression of PRPS1 among different age groups using data from the Tumor Neuroblastoma Public-Versteeg 88 database. (**C**) The expression of PRPS1 in recurrent and non-recurrent patients using data from the Tumor Neuroblastoma Public-Versteeg 88 database. (**D**) The expression of PRPS1 in live or dead patients using data from the Tumor Neuroblastoma Public-Versteeg 88 database. (**E**) Real-time qPCR analysis of PRPS1 mRNA expression levels in four neuroblastoma cell lines: AS, BE(2)-C, DZ, and SHEP1. (**F**) Western blot analysis of PRPS1 expression in the four aforementioned neuroblastoma cell lines. α-tubulin was used as the loading control.

To investigate whether PRPS1 is associated with neuroblastoma patient prognosis, a Kaplan–Meier analysis based on the R2 database was first conducted. A very close relationship was observed between PRPS1 expression and neuroblastoma prognosis. Specifically, when PRPS1 was highly expressed, the patient’s prognosis was poor, and the corresponding survival rate was low. However, when PRPS1 expression levels were low, the patients had a good prognosis and a high survival rate (Figure 1A). The expression level of PRPS1 was low when a patient was diagnosed at less than 18 months of age. In contrast, diagnosed patients that were older than 18 months exhibited significantly increased PRPS1 expression levels (Figure 1B). Neuroblastoma tumors have a very high recurrence rate [29]. Consequently, the recurrence rates for patients were evaluated using the R2 database. PRPS1 expression levels were significantly higher in patients with relapse. In contrast, patients that did not relapse had low PRPS1 expression levels, indicating that PRPS1 was closely associated with the recurrence of neuroblastoma (Figure 1C). Also, the expression levels of PRPS1 were higher in patients that died of neuroblastoma (Figure 1D). Thus, these results indicated that higher degrees of tumor malignancy was associated with higher PRPS1 expression and that a close relationship existed between PRPS1 expression and neuroblastoma prognosis.

Following the above analyses, PRPS1 expression levels were evaluated in four neuroblastoma cell lines using qRT-PCR and Western blotting: SK-N-AS, BE(2)-C, SK-N-DZ, and SHEP1 (Figure 1E,F). These analyses confirmed that PRPS1 was commonly expressed in neuroblastoma cells.

### 3.2. Down-Regulated PRPS1 Inhibits Proliferation of Neuroblastoma Cells

Three types of neuroblastoma cells can be observed in vitro: neuroblastic (N-type), substrate-adherent (S-type), and intermediate (I-type). Among them, I-type cells can simultaneously express cell markers of either N- or S-type cells and can also form clones on nude mice and semi-solid medium. Therefore, I-type cells are considered a population of neuroblastoma stem cells or malignant neural crest stem cells [30]. As the prognosis of N- and I-type cells is poor, and that of S-type cells is good, we chose one I–type (BE(2)-C) neuroblastoma cell and one S-type (SHEP1) neuroblastoma cell for the investigation [31,32].

**Figure 2:**
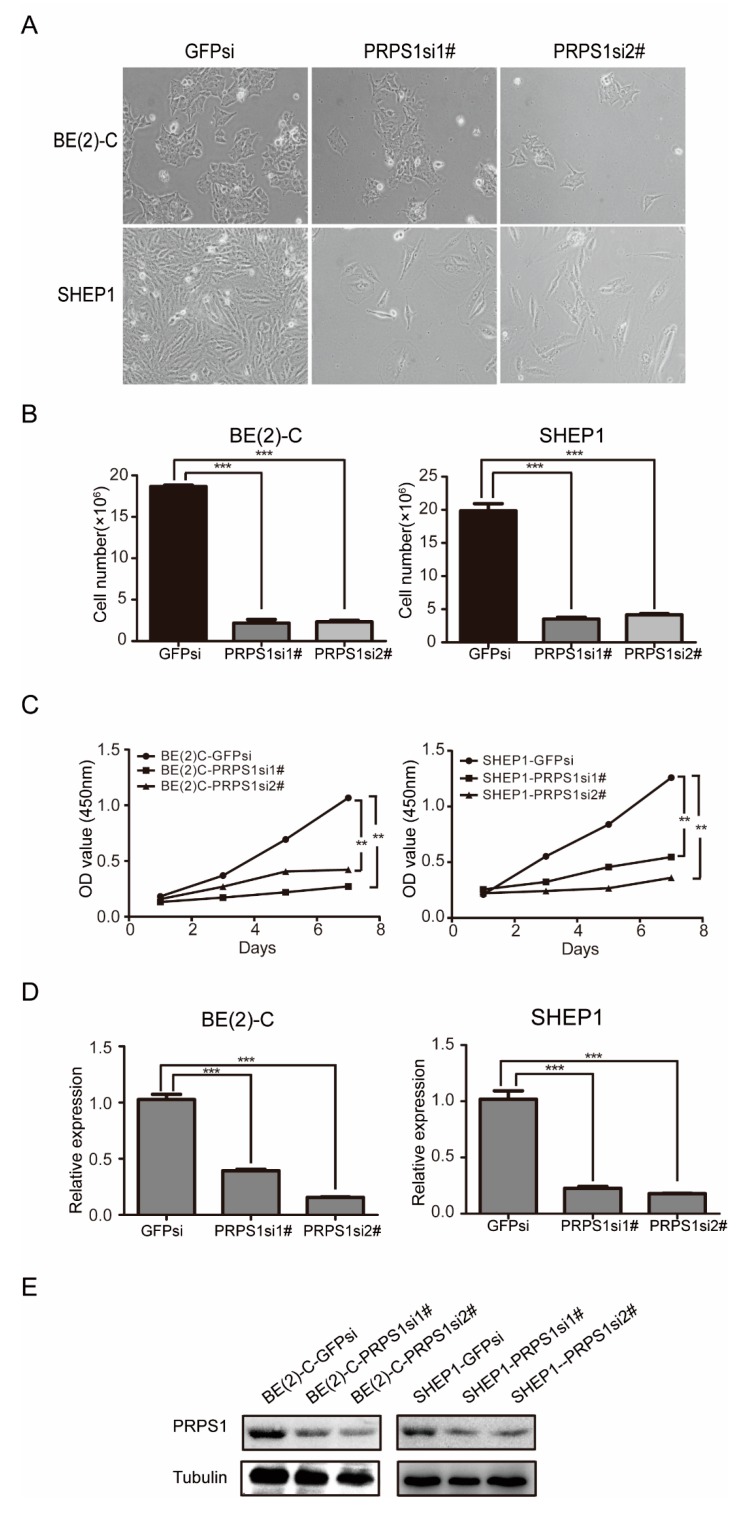
Down-regulation of PRPS1 inhibits neuroblastoma cell proliferation. (**A**) Morphological examination of neuroblastoma cells with either a GFP or PRPS1 knockdown. Scale bar: 20 µm. (**B**) Cellular enumeration with trypan blue dye staining. (**C**) Neuroblastoma cell growth curves with either a GFP or PRPS1 knockdown, as indicated by CCK-8 (Cell Counting Kit-8) assays. (**D**) Real-time qPCR analysis of PRPS1 mRNA expression levels in neuroblastoma cells with either a GFP or PRPS1 knockdown. (**E**) Western blot analysis of PRPS1 expression in neuroblastoma cells with either a GFP or PRPS1 knockdown. α-tubulin was used as the loading control. The data shown in panels B–D represent averages obtained from three independent experiments, and the data are presented as the mean ± S.D. (error bars). Statistical analyses were performed using two-tailed Student’s *t*-tests, *** *p* < 0.001, ** *p* < 0.01. OD: optical density; S.D.: standard deviation.

To explore the biological role of PRPS1 in cell proliferation of neuroblastoma cells, we knocked down PRPS1 using a lentivirus interference system in the BE(2)-C and SHEP1 cell lines (Figure 2A,B). Cell growth curve experiments using the CCK-8 method were then used to evaluate cell viability and proliferation of the experimental (PRPS1si) and control (GFPsi) group cells (Figure 2C–E). The data demonstrated that the proliferative capacity of the experimental group was significantly lower than that of the control group, suggesting that PRPS1 was positively involved in the proliferation of neuroblastoma cell lines BE(2)-C and SHEP1.

### 3.3. Down-Regulated PRPS1 Disturbs DNA Synthesis in Neuroblastoma Cells

**Figure 3:**
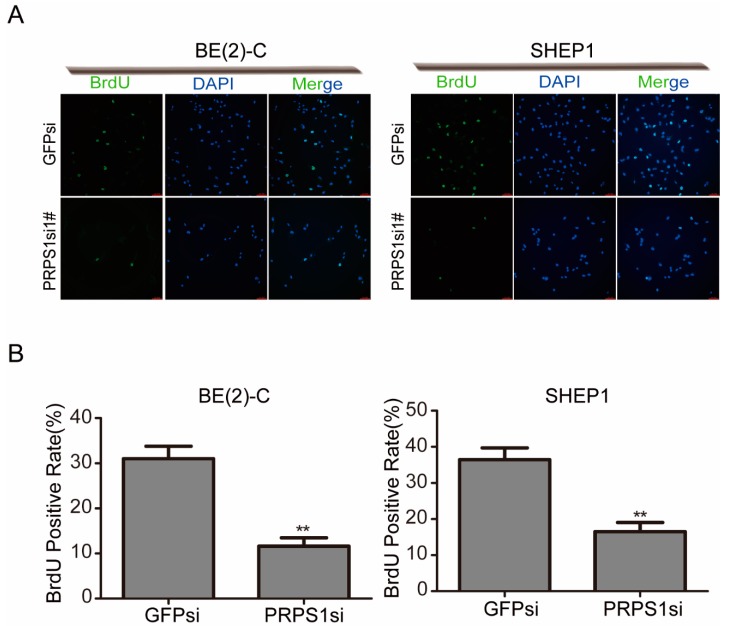
Down-regulation of PRPS1 inhibits BrdU (bromodeoxyuridine) incorporation of neuroblastoma cells. (**A**) Neuroblastoma cells with either a GFP or PRPS1 knockdown that were grown on 24-well culture plates and treated with BrdU for 30 min. Cells were then stained with the BrdU antibody (green) and counterstained with DAPI (blue). Scale bar: 20 µm. (**B**) The abundance of BrdU-positive cells was determined microscopically at 20×. Values in panels B represent the averages obtained from three independent experiments, and the error bars show S.D. Statistical analyses were performed using two-tailed Student’s *t*-tests, ** *p* < 0.01.

Because the changes in proliferation are often associated with disturbances in DNA synthesis, we measured rates of incorporation of BrdU in cell cultures. BrdU was used to label cells, which resulted in a significantly reduced proportion of BrdU-positive cells in the experimental group due to down-regulation of PRPS1 (Figure 3A,B). The result showed that the DNA synthesis of cells was significantly blocked after PRPS1 silencing.

**Figure 4:**
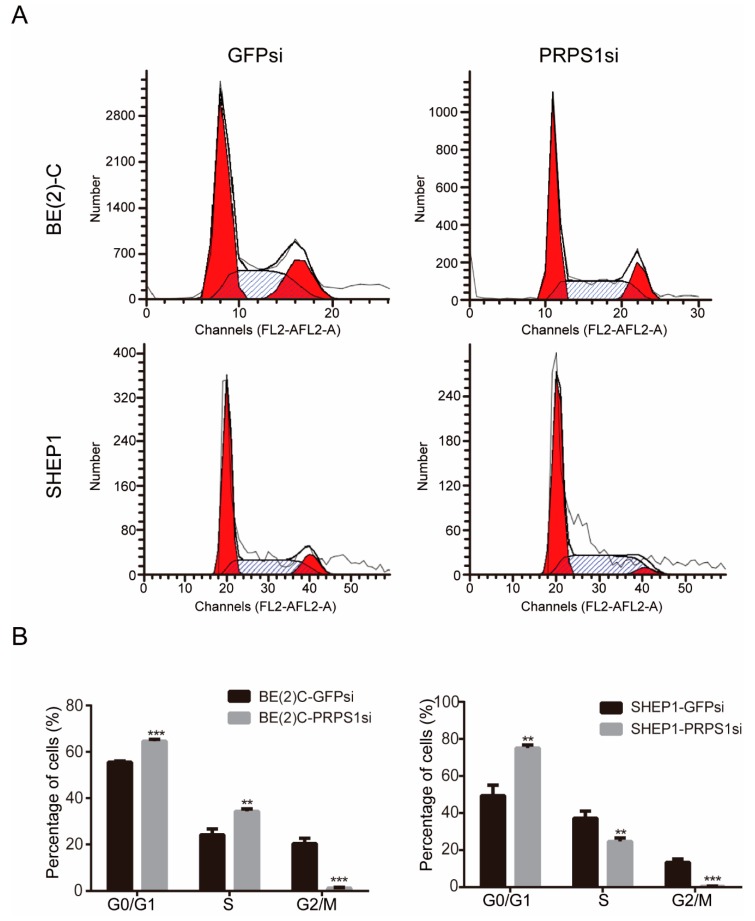
Down-regulation of PRPS1 arrests the cell cycle of neuroblastoma cells. (**A**) Flow cytometry analysis of the cell cycle distributions. (**B**) Statistical analysis of the cell cycle phase distribution. Values in panels B represent the averages obtained from three independent experiments, and the error bars show S.D. Statistical analyses were performed using two-tailed Student’s *t*-tests, *** *p* < 0.001, ** *p* < 0.01.

The abovementioned data showed that down-regulated PRPS1 inhibited neuroblastoma cell proliferation. As reported, cell proliferation is often associated with cell cycle progression. Thus, FCM was used to investigate the cell cycle of neuroblastoma cells in relation to PRPS1 expression. The cell cycles of BE(2)-C and SHEP1 cells significantly changed after interference with PRPS1 gene expression. Specifically, the proportion of cells in the G0/G1 phase significantly increased, the proportion of cells in the S phase showed a variable change depending on the cell type, and the proportion of cells in the G2/M phase also significantly decreased (Figure 4A,B).

These results indicated that PRPS1 played an important role in maintaining neuroblastoma proliferation, and that down-regulating PRPS1 led to an increase in the proportion of cells in the G0/G1 phase accompanied by a decrease in the efficiency of DNA synthesis.

### 3.4. PRPS1 Inhibition Decreases Tumorigenicity of Neuroblastoma Cells In Vitro and In Vivo

**Figure 5:**
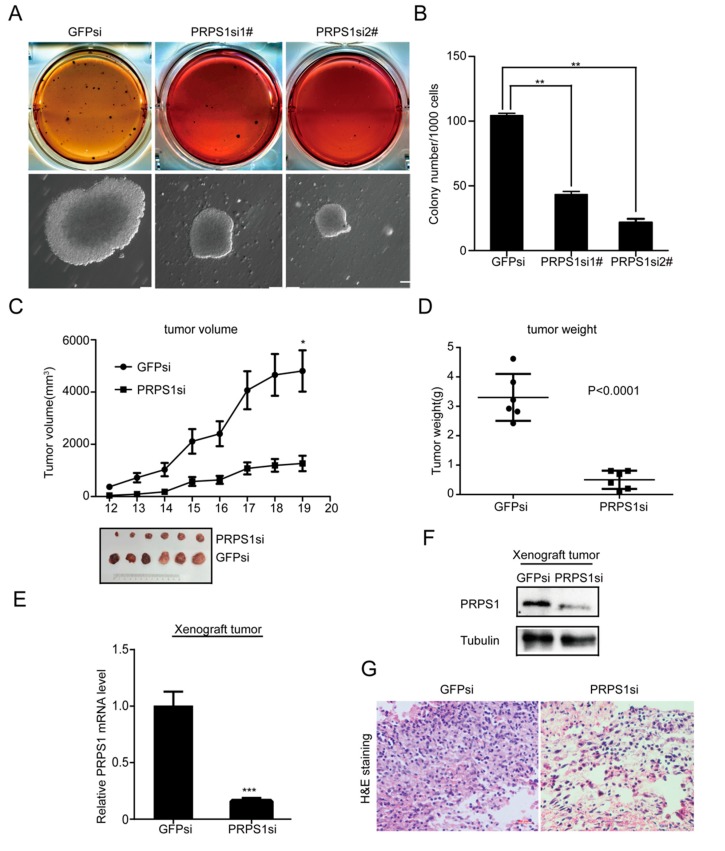
Down-regulation of PRPS1 suppresses colony formation in vitro and tumor growth in vivo. (**A**) BE(2)-C cells with either a GFP or PRPS1 knockdown were plated at a concentration of 1 × 10^3^ cells per well in six-well culture plates. After 14 to 21 days of culture, soft agar colonies grew from cells with the knockdowns, and the colonies were microscopically imaged. Scale bar: 50 µm. (**B**) The colonies were stained with MTT for enumeration. Colonies that were >0.5 mm in diameter or contained >50 cells were recorded. (**C**) Tumor growth in NOD/SCID (non-obese diabetic/severe combined immunodeficient) mice harboring BE(2)-C cells with GFP or PRPS1 knockdowns, and measurement of xenograft tumor volumes using calipers. (**D**) Xenograft tumor weights were measured after tumor removal. (**E**) Real-time qPCR analysis of PRPS1 mRNA expression levels in xenograft tumors. (**F**) Western blot analysis of PRPS1 expression in xenograft tumors. α-tubulin was used as the loading control. (**G**) Histological analysis of xenograft tumor tissues in the xenograft tumors. Data in panels B-E represent the averages obtained from three independent experiments, and the error bars show S.D. Statistical analyses were performed using two-tailed Student’s *t*-tests, *** *p* < 0.01, ** *p* < 0.05.

We next investigated the effect of down-regulating PRPS1 on neuroblastoma cell self-renewal in BE(2)-C cells via soft agar clonogenic assays. The cells were plated at a level of 1 × 10^3^ cells per well (six-well culture plates), allowed to grow for two to three weeks, and then examined microscopically. Knockdown of PRPS1 in BE(2)-C cells resulted in significantly fewer clones than in the control group (Figure 5A), which was consistent with the prior statistical analysis (Figure 5B). These results indicated that when PRPS1 gene expression was disrupted in BE(2)-C cells, the ability of cells to self-renew was suppressed.

NOD/SCID mice were then used to establish a mouse model. Mice were injected with either PRPS1si or GFPsi BE(2)-C cells, and subcutaneous solid tumors were allowed to form, followed by measurement of tumor volumes. The PRPS1si group cells exhibited significantly lower growth rates than those in the GFPsi group (Figure 5C). Dissection and weighing of subcutaneous tumor tissues indicated significantly lower weights in the PRPS1si group than in the GFPsi group (Figure 5D). qRT-PCR and Western blot analysis of PRPS1 expression in tumor tissues further revealed that PRPS1 expression levels in tumor tissues were consistent with cellular level results (Figure 5E,F). Hematoxylin-eosin staining of the dissected tumors also revealed bright blue hematoxylin staining of nuclei, while cytoplasms were stained pink from eosin. The nucleoplasmic ratio of tumors in the experimental group was slightly higher than that of the GFPsi group. Furthermore, the degree of tightness between cells was significantly lower in the experimental group than in the GFPsi group, indicating that PRPS1 was involved in the regulation of the tumorigenic ability of neuroblastoma (Figure 5G). These results are consistent with previous research into GBM [23] and in colorectal cancer (CRC) [33].

### 3.5. PRPS1 Expression Is Consistent with MYCN Amplification in Neuroblastoma Patient

The tumor proto-oncogene MYCN is an important oncogene in the pathogenesis of neuroblastoma [34]. It was first identified in neuroblastoma cells, and about 22% of neuroblastoma patients have abnormal amplification in MYCN [35]. Abnormal amplification of MYCN is considered an important indicator of poor prognosis for neuroblastoma [36,37,38,39,40]. The correlation between PRPS1 expression levels and proto-oncogene MYCN amplification was thus subsequently evaluated.

**Figure 6:**
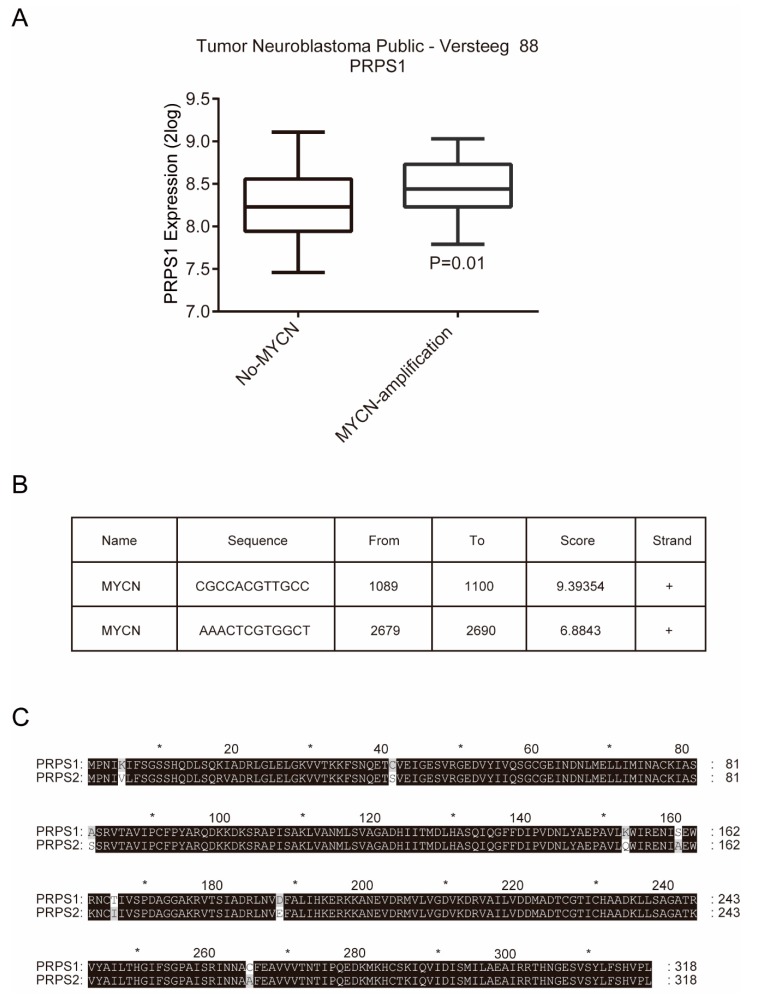
PRPS1 is closely related to MYCN in neuroblastoma patient. (**A**) The relationship between PRPS1 expression and MYCN amplification, based on data from the R2 platform. (**B**) The potential MYCN binding sites in human PRPS1 gene, based on data from the JASPAR database. (**C**) The amino acid sequence conservation of PRPS1 and PRPS2.

Based on the R2 database, we performed bioinformatics analysis and found that the expression of PRPS1 was significantly higher in patients with MYCN amplification than in patients without MYCN amplification (Figure 6A). To find a direct role of MYCN in the regulation of PRPS1 expression, an online transcription factor binding profiles analysis based on the JASPAR database (http://jaspar.genereg.net) was used to determine whether there was a direct binding of MYCN to the promoters of PRPS1 gene. Analysis performed on the sequences 0–3 kb upstream from the transcription start sites of PRPS1 gene identified two MYCN binding sites (Figure 6B). Previous evidence indicated that silencing MYC significantly reduced both mRNA and protein levels of PRPS1 in brain tumor-initiating cells, and chromatin immunoprecipitation assay showed that MYC interacted with the promoter regions of PRPS1 gene [41]. These results suggested that MYCN regulates the expression of PRPS1 directly by binding to its promoter regions.

Phosphoribosyl pyrophosphate synthetase 2 (PRPS2) and PRPS1 belong to PRPS enzyme family, and they share approximately 95% amino acid homology (Figure 6C) [42]. As reported, PRPS2 also involves in cancer cell progress. PRPS2 expression is correlated with cell apoptosis in TM4 Sertoli cells [43]. Scientists have also found evidence of the interaction between MYC and PRPS2. Xue et al. found that PRPS2 is regulated by MYCN [44]. Mannava et al. found that PRPS2 gene is a direct C-MYC target [45]. PRPS1 and PRPS2 have interchangeable roles in normal cells [46], and they may exhibit similar functions. Overall, we boldly speculate that PRPS1 is closely related with MYCN to regulate cell proliferation in neuroblastoma.

## 4. Discussion

Neuroblastoma is an extracranial tumor disease that is one of the most common malignant solid tumors in children under the age of one; it accounts for approximately 5% of all cancer diagnoses in pediatrics [9,47].

Herein, we determined that PRPS1 expression was a potential prognostic marker in neuroblastoma patients. As a key enzyme in purine metabolism, PRPS1 catalyzes the first step in nucleotide synthesis. In our experiments, high PRPS1 expression was associated with poor outcomes, and low expression was associated with a good prognosis. Moreover, PRPS1 expression was associated with patient age, recurrence, and cause of death in neuroblastoma patients. In general, PRPS1 was closely related to poor neuroblastoma prognosis.

To confirm the expression level of PRPS in neuroblastoma cells, we extracted total cellular RNA and subjected it to a qRT-PCR assay, and then used total cellular protein to perform a Western blot analysis. We found that PRPS1 was commonly expressed in neuroblastoma cells.

Down-regulation of PRPS1 expression resulted in significantly lower cell viability and proliferation abilities. Additionally, BrdU assays showed that DNA synthesis of cells was significantly blocked after PRPS1 silencing, suggesting that cell proliferation was inhibited via a decrease in DNA synthesis efficiency. Therefore, using FCM, we further analyzed cell cycle status in BE(2)-C and SHEP1 cells with or without PRPS1 knockdown. We found that silencing PRPS1 caused the proportion of cells in the G0/G1 phase to increase significantly, and bioinformatics analysis revealed the close relationship between PRPS1 and the cell cycle regulatory pathway. Consistent with the results shown in SHEP1 cells, silencing of *PRPS1* genes had significant effects on the entrance of cells into the S phase in human fibroblasts [48]. Jing et al. explored the role and activation mechanism of PRPS1 in cell cycle progression of CRC and observed a peak in its enzymatic activity during S phase [33]. The process of the cell cycle is ordered and rigorous, which involves doubling of DNA and other cellular contents to duplicate a cell. Cell cycle checkpoint is the mechanism in the cell cycle that ensures the quality of DNA replication and chromosome distribution. It is a type of negative feedback adjustment mechanism. Once abnormal events occur during cell cycle progressions, such as the block of DNA damage or DNA replication, the checkpoint signaling pathway is activated immediately to interrupt the cell cycle. After the cell is repaired or rectified, the cell cycle can resume operation [49]. In BE(2)-C cells with PRPS1 knockdown, there was an increase in the proportion of cells in the S phase. We speculated that the reduced BrdU uptake rate would trigger an S-phase checkpoint, which would lead to an accumulation of cells in S-phase because it would take longer for them to complete the replication of the whole genome, but more evidence and further research are needed.

Interestingly, PRPS1 may play different roles in different cancers. Cell proliferation is often associated with cell cycle progression, and apoptosis can also reduce cell proliferation and inhibit tumor growth. Jing et al. have found that loss of PRPS1 phosphorylation delayed the cell cycle and decreased cell proliferation in CRC [33]. Meanwhile, Ma et al. have pointed out that silencing PRPS1 had no significant effect on cell cycle regulators but only increased cell apoptosis in B-cell acute lymphoblastic leukemia (B-ALL) cell lines [25]. Besides, silencing PRPS1 can increase cell apoptosis in human breast cancer cells [50] and GBM [23]. This suggests that we should further study changes in PRPS1 knockdown in neuroblastoma in the future.

In this study, our data also revealed that mice implanted with PRPS1 knockdown neuroblastoma cells exhibited reduced tumor volume and weight. In their xenograft mouse model, Li et al. also found dramatic decreases in tumor volume and prolonged survival time in their PRPS1si group in GBM [23]. Jing et al. also found increased PRPS1 activity, which is important to promoting tumorigenesis; this is consistent with our findings [33].

Using bioinformatics analysis to explore the mechanism by which PRPS1 regulates cell proliferation, we found that PRPS1 expression was significantly higher in patients with MYCN amplification. MYCN is an important proto-oncogene [51], whose amplification is abnormal in more than one-fifth of neuroblastoma patients. Based on the JASPAR database, we have identified two potential MYCN binding sites in human PRPS1 gene. Studies have already found that MYC has a direct regulatory relationship with PRPS1 [41]; moreover, some studies have found the same for PRPS2 [44,45]. As we have shown, PRPS1 and PRPS2 have similar amino acid structures and may exhibit similar functions in neuroblastoma cells. Despite PRPS1 and PRPS2 being widely expressed in all body tissues, their expression levels significantly differ. PRPS1 is highly expressed in the brain and adrenal gland tissues that exhibit slow or little proliferation. In contrast, PRPS2 is highly expressed in rapidly proliferating tissues, including those of the thymus, lungs, stomach, small intestine, spleen, and testicles [52]. Neuroblastoma is a brain tumor; therefore, we speculate that PRPS1 works closely with MYCN to regulate cell proliferation in brain tumor neuroblastoma.

Taken together, the results of our study demonstrate that PRPS1 is essential to the regulation of neuroblastoma cell proliferation and tumorigenesis, though the specific mechanism remains unclear. Developing therapeutics against PRPS1 could synergize with radiation and chemotherapy to achieve improved management of neuroblastoma.
